# Innate immunity is a key factor for the resolution of inflammation in asthma

**DOI:** 10.1183/09059180.00012514

**Published:** 2015-03

**Authors:** Cindy Barnig, Bruce D. Levy

**Affiliations:** 1 Dept of Chest Disease, University Hospital of Strasbourg and FMTS (Fédération de Médecine Translationnelle de Strasbourg), Strasbourg, France; 2 Pulmonary and Critical Care Medicine, Dept of Internal Medicine, Brigham and Women’s Hospital and Harvard Medical School, Boston, MA, USA

## Abstract

The resolution of inflammation is an integral and natural part of the physiological response to tissue injury, infection and allergens or other noxious stimuli. Resolution is now recognised as an active process with highly regulated cellular and biochemical events. Recent discoveries have highlighted that innate inflammatory cells have bimodal effector functions during the inflammatory response, including active roles during the resolution process. Several mediators displaying potent pro-resolving actions have recently been uncovered. Lipoxin A_4_, the lead member of this new class of pro-resolving mediators, has anti-inflammatory actions on type 2 innate lymphoid cells and pro-resolving actions through natural killer cells in asthma immunobiology. Eosinophils are also able to control crucial aspects of resolution through the generation of pro-resolving mediators. Uncontrolled asthma has been associated with a defect in thegeneration of specialised pro-resolving mediators, including lipoxin A_4_ and protectin D1. Thus, bioactive stable analogue mimetics of these mediators that can harness endogenous resolution mechanisms for inflammation may offer new therapeutic strategies for asthma and airway inflammation associated diseases.

## Introduction

Asthma is characterised by increased and chronic airway inflammation, with mucosal infiltration of inflammatory cells and release of pro-inflammatory cytokines and lipid mediators [1]. The airway inflammation of asthma, which is often allergic by nature, has been attributed to ongoing adaptive helper T-cell type-2-mediated inflammation [2]. There is increasing evidence that innate immunity plays critical roles in the pathobiology of asthma, in chronic stable inflammation and during episodes of exacerbated acute inflammation in response to a variety of stimuli, such as allergen inhalation, exposure to environmental pollutants or microbial infection [3].

Most studies have focused on the role of innate inflammatory cells (*i.e.* eosinophils, mast cells, basophils, neutrophils, macrophages, several different subsets of dendritic cells, and newly described innate lymphoid cells (ILCs)) along with activated resident structural cells (epithelial cells, fibroblasts and airway smooth muscle cells) to accentuate and perpetuate the airway inflammation in asthma. Indeed, these cells release a vast array of pro-inflammatory and potentially tissue destructive compounds (eicosanoids, reactive oxygen species, cytokines, chemokines, growth factors and proteases) into the extracellular space [4]. Recent discoveries have highlighted that many innate inflammatory cells have bimodal effector functions during the inflammatory response, with some having active roles during the resolution process.

Resolution of inflammation in asthma is characterised by clearance of inflammatory leukocytes from the lung, restoration of epithelial barrier function and dampening of airway hyperreactivity [5]. During resolution, multiple specialised mediators and cellular mechanisms are enlisted to generate endogenous “braking signals” to restore tissue homeostasis [6]. Several classes of counter-regulatory lipid mediators have been recently discovered that are generated from polyunsaturated fatty acids (PUFAs) during inflammation to promote resolution [7]. These specific pro-resolving lipid mediators are produced *via* biosynthetic circuits engaged during cell–cell interactions between different innate immune cells and structural cells at sites of inflammation in the lung and have a large array of anti-inflammatory and pro-resolving actions, including on the newly described ILCs [8].

In this article, we discuss recent studies on the role of pro-resolving lipid mediators in asthma inflammation with a focus on ILCs and eosinophils.

## Inflammatory responses and the resolution of inflammation

Acute inflammation is an indispensable host response to insult or tissue injury and is initiated within minutes of recognition of a danger signal [9]. The acute inflammatory process is characterised by rapid recruitment of granulocytes (*i.e.* neutrophils, eosinophils and basophils) to the inflammatory site, the relative contributions of these cell types are dependent on the nature and the location of the inflammatory response. The initial events of acute inflammation are coordinated by many pro-inflammatory mediators (*i.e.* lipid mediators such as prostaglandins and leukotrienes, cytokines, and chemokines) that regulate vascular permeability and initial recruitment of leukocytes [10].

In health, the acute inflammatory response is generally self-limited, resolving within hours or days; however, in many human diseases, including asthma, resolution fails and inflammation stalls for a prolonged period. Therefore, failure to adequately resolve acute inflammation in asthma may contribute to chronic changes in airway structure and function causing clinical expression of asthma symptoms (reviewed in [11]).

Natural resolution of inflammation is now recognised an active host response. While it is driven, in part, by decrements in pro-inflammatory mediators, the promotion of resolution involves early signalling pathways engaging biosynthetic circuits for the later formation of counter-regulatory mediators [12]. For effective resolution of inflamed tissues to occur cessation of the recruitment of granulocytes is required, followed by the recruitment of monocytes that differentiate into macrophages, which clear inflammatory cells and tissue debris, leading ultimately to the restoration of tissue structure and function [13]. During this process, tissue granulocytes undergo apoptosis, a highly regulated cell death mechanism that prevents the release of histotoxic cellular contents [14]. Clearance of apoptotic neutrophils prompts a switch from a pro-inflammatory to an anti-inflammatory macrophage phenotype, which is a prerequisite for macrophage egress *via* the lymphatic vessels favouring a return to tissue homeostasis [15]. Clearance of apoptotic neutrophils also leads to the production of additional mediators that suppress the progression of inflammation and promote repair of damaged tissues [16, 17].

While several classes of mediators participate in resolution, the enzymatic transformations of PUFAs to specific pro-resolving agonists are of particular interest. These PUFA-derived mediators display cell-type selective anti-inflammatory, pro-resolving, anti-fibrotic, anti-angiogenic and anti-infective actions [7, 18].

## PUFAs derived pro-resolving mediators

The use of experimental models of acute inflammation that naturally resolve (*i.e.* self-limited return to homeostasis) has led to the identification of a novel family of lipid mediators generated from PUFAs, named lipoxins, resolvins, protectins and maresins. These endogenous counter-regulatory mediators actively stimulate cardinal signs of resolution, namely, cessation of leukocytic infiltration, counter-regulation of pro-inflammatory mediators, and the uptake of apoptotic neutrophils and cellular debris (reviewed in [7]).

**FIGURE 1:**
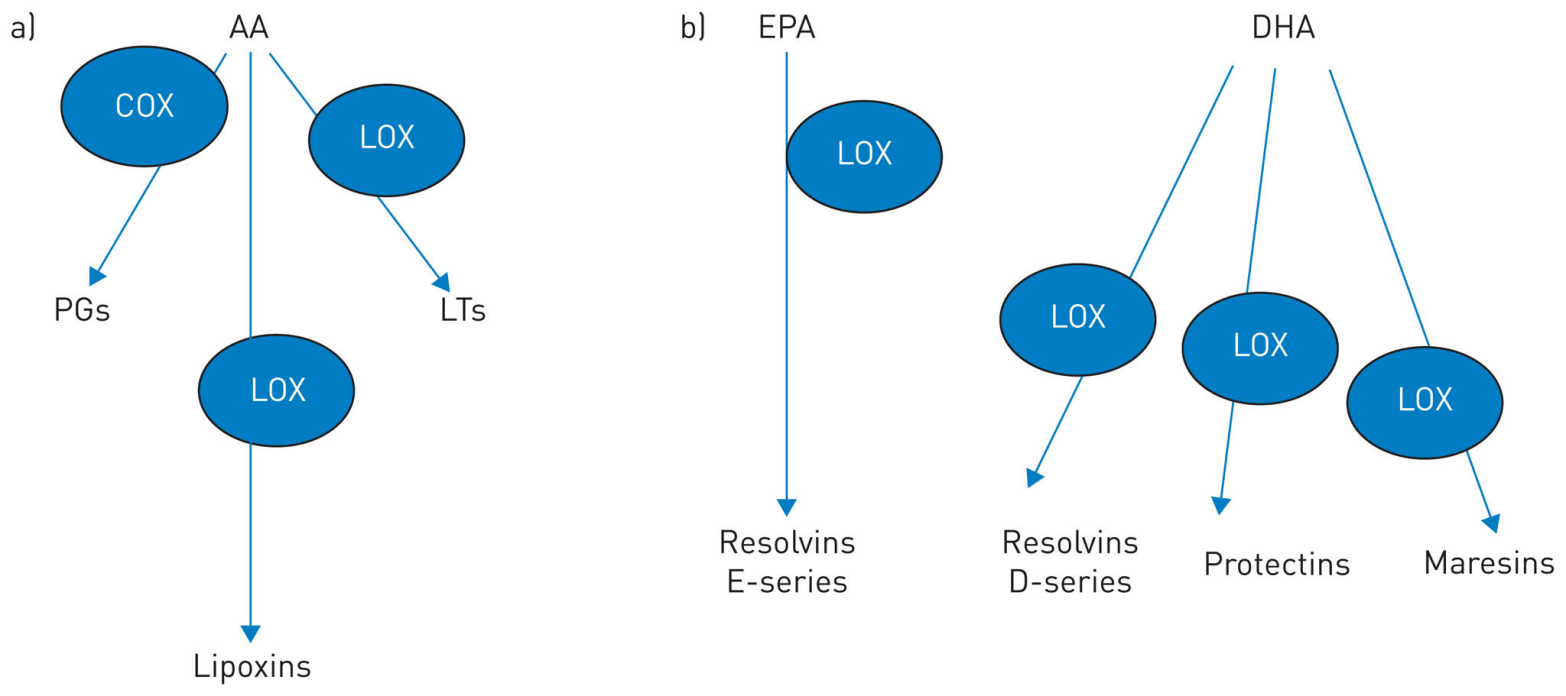
Formation of bioactive lipid mediators. Specific pro-resolving lipid mediators are enzymatically derived from host essential polyunsaturated fatty acids (PUFAs), including a) the omega-6 PUFA arachidonic acid (AA) (C20:4n-6), and b) the omega-3 PUFAs eicosapentaenoic acid (EPA) (C20:5n-3) and docosahexaenoic acid (DHA) (C22:6n-3) *via* lipoxygenase (LOX) pathways. AA also forms a range of pro-inflammatory mediators, such as prostaglandins (PGs) *via* cyclooxygenase (COX)-2, and the leukotrienes (LTs) *via* multiple LOX actions.

The omega-6 PUFA, arachidonic acid (20:4n-6) is incorporated into cellular phospholipids, and upon cell activation, specific phospholipase A_2_ enzymes release arachidonic acid from the sn-2 fatty acyl bond of phospholipids. Arachidonic acid can then be converted enzymatically by cyclooxygenase (COX) to prostaglandins, by 5-lipoxygenase (LOX) to leukotrienes, or by 5-LOX in collaboration with 12-LOX or 15-LOX to lipoxins [19]. The omega-3 PUFAs eicosapentaenoic acid (EPA) (20:5n-3) and docosahexaenoic acid (DHA) (22:6n-3) can be enzymatically transformed *via* LOX pathways to resolvins, protectins and maresins (fig. 1) [20–22]. The enzymatic generation of these families occurs primarily *via* transcellular biosynthesis, and in some cases within a single cell-type.

During the acute inflammatory response, the biosynthesis of these resolution-phase mediators is initiated during lipid-mediator class switching, in which production of the classic initiators of acute inflammation, prostaglandins and leukotrienes, switches to specialised pro-resolving mediators as inflammation resolves [23].

These mediators display receptor-mediated cell type specific actions and display potencies in the low nanomolar range (table 1).

### Lipoxins

Arachidonic acid-derived lipoxin A_4_ (LXA_4_) was the first PUFA-derived mediator found to have anti-inflammatory and pro-resolving activities [59]. Lipoxins are derived from the sequential actions of LOXs and are principally generated *via* biosynthetic circuits engaged during cell–cell interactions at sites of inflammation. Although lipoxins are present in low abundance during the initiation of acute inflammation, their levels increase substantially during resolution [23, 60]. In the lung, 15-LOX is a key enzyme for lipoxin generation and is expressed by many cells in the inflamed lung, including bronchial epithelial cells, macrophages and eosinophils [61–64].

Lipoxins act locally and then are rapidly inactivated by metabolic enzymes *via* pathways shared with other eicosanoids [18, 65]. In addition, lipoxin epimers can be generated in the presence of aspirin (acetylsalicylic acid) that are longer acting because of a reduced rate of inactivation [26, 66].

LXA_4_ is an agonist for ALX/FPR2 receptors, which are expressed on both human airway epithelial cells and leukocytes [67]. In addition, ALX/FPR2 receptors can be induced by specific inflammatory mediators [68]. ALX/FPR2 receptors are also expressed on natural killer (NK) cells and type 2 ILCs [31]. In addition to lipoxins signalling through ALX/FPR2, these small molecules can act as antagonists at cysteinyl leukotriene receptors and can also signal *via* the aryl hydrocarbon receptor (AHR) [69].

Lipoxins demonstrate cell type-specific actions *in vitro* relevant to asthma immunobiology (table 1). These actions include inhibition of granulocyte locomotion, shape change and transmigration, and degranulation and stimulation of monocyte chemotaxis and macrophage engulfment of apoptotic neutrophils. In addition to this leukocyte-specific activity, lipoxins promote restoration of injured airway epithelium by indirectly blocking the release of the pro-inflammatory cytokines interleukin (IL)-6 and IL-8 by the epithelium [39].

### EPA and DHA derived pro-resolving mediators

Population surveys report that diets rich in omega-3 fatty acids are associated with lower asthma prevalence [70]. Recent studies have identified a new family of pro-resolving lipid mediators generated from the omega-3 fatty acids, EPA (20:5n-3) and DHA (22:6n-3) [20]. These include the EPA-derived E-series resolvins (RvE1 and RvE2), the DHA-derived D-series resolvins (RvD1–D6), neuroprotectin/ protectin, and maresin (fig. 1) [71].

Resolvins and protectins bear similaritiy to lipoxins in that their epimers can also be generated by the “alternative” acetylated COX-2 pathway in the presence of aspirin, thus producing “aspirin-triggered” forms [21].

These mediators, like lipoxins, act as potent anti-inflammatory lipid mediators limiting neutrophil influx, and also promote resolution of inflammation by stimulating the clearance of apoptotic cells and inflammatory debris by macrophages (table 1) [72]. RvE1 serves as an agonist at CMKLR1 receptors [50]. CMKLR1 is expressed on monocytes/macrophages and plasmacytoid dendritic cells [73–75]. CMKLR1 is also expressed on NK cells and type 2 ILCs [31].

### PUFA derived pro-resolving mediators in allergic airway inflammation and asthma

The biological activity of the lipoxins in asthma and allergic disease has been defined over the past two decades (table 2). Bioactive, LXA_4_ stable analogues have been prepared that block airway hyperresponsiveness and allergic inflammation in animal models, including eosinophil trafficking and tissue accumulation [68, 76]. They also block oedema formation and reduce the levels of the pro-inflammatory mediators IL-5, IL-13, CCL11, prostanoids and cysteinyl leukotrienes [68]. In humans, LXA_4_ is generated during asthmatic responses [78–80] and, when administered to asthmatic subjects *via* nebulisation, LXA_4_ attenuates leukotriene C_4_-induced bronchoconstriction [81].

More severe variants of asthma are associated with diminished lipoxin biosynthesis compared with milder asthma [79, 82], suggesting that the chronic inflammatory response in asthma may be due, in part, to defective generation of pro-resolving mediators leading to inadequate counter-regulation (table 3). Decreased formation of lipoxins in uncontrolled asthma has now been identified in distinct populations of adults and children from several countries [33, 86, 87, 89]. There is also a decrease in lipoxins in the lungs of patients with aspirin-intolerant asthma [83], and exercise-induced asthma compared with the lungs of healthy persons [87].

RvE_1_ is present in the lung [95]. In an experimental model of asthma, RvE_1_ dampens the development and promotes the resolution of allergic airway responses [60, 96, 97]. RvD_1_ and aspirin-triggered RvD_1_also promote resolution of allergic airways responses [54]. Protectin D1 (PD1) mediates bronchoprotective actions in a murine experimental model of allergic lung inflammation [77] and is decreased in exhaled breath condensates during acute exacerbations of asthma [77].

## ILCs are newly identified players in asthmatic inflammation and targets for specialised pro-resolving mediators

ILCs comprise a newly described family of haematopoietic effectors that play protective roles in innate immune responses to infectious microorganisms, lymphoid tissue formation, tissue remodelling after damage inflicted by injury or infection, and the homeostasis of tissue stromal cells (reviewed in [98]).

### NK cells

NK cells are prototypical members of the ILC family. NK cells serve essential roles in host defence, including cytokine secretion, contact-dependent cell–cell signalling and direct killing of other immune cells, and are involved in combating tumours, viral infections, parasites and bacteria [99]. Roles for NK cells in asthma and allergic diseases are undefined and both disease-promoting and disease-controlling functions have been suggested [100].

Potential roles for NK cells in resolution of allergic airway responses have been recently defined. In a murine model of allergic lung inflammation, NK cells accumulate during resolution in the lung draining lymph nodes [97]. Depleting NK cells at the peak of the inflammatory response after allergen challenge delays clearance of airway eosinophils and antigen-specific CD4^+^ T lymphocytes [97]; however, depletion of NK cells before allergen challenge has been shown to inhibit airway eosinophilia [101, 102]. Therefore, the timing of depletion of NK cells during the allergic inflammatory response may reveal different functions of NK cells during inflammation.

Co-culture experiments revealed that human NK cells could induce neutrophil [103] and eosinophil apoptosis [31, 104]. Moreover, NK cells from severe asthmatic subjects have a reduced capacity to augment eosinophil apoptosis [31]. Apoptosis of inflammatory cells is a non-inflammatory mechanism of cell removal and plays a critical role in successful resolution of the inflammatory response. Neutrophils and eosinophils can release toxic substances and enzymes harmful not only to pathogens, but also to surrounding tissue. In asthma, timely regulation of eosinophil activation and apoptosis is probably crucial to avoid tissue damage and induce resolution of inflammation [105]. Apoptotic granulocytes can subsequently be removed by tissue macrophages before their toxic contents leak out into the tissue and result in extensive damage. By accelerating granulocyte apoptosis, NK cells may play a role in limiting the inflammatory response and may be involved in the resolution of acute inflammation.

**FIGURE 2:**
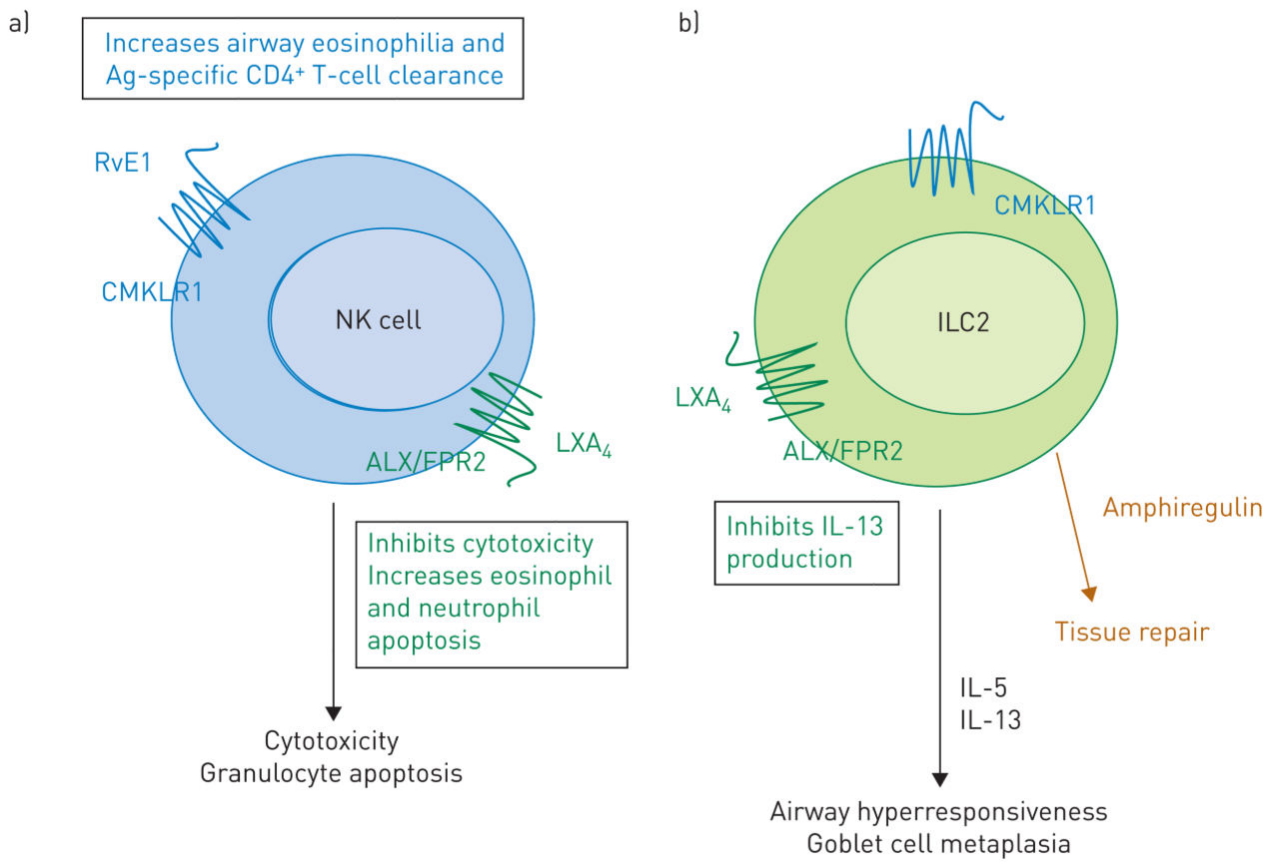
Key functions of innate lymphoid cells in resolution of inflammation in asthma. a) Natural killer (NK) cells and b) type 2 innate lymphoid cells (ILC2s) express the pro-resolving receptors ALX/FPR2 for lipoxin A_4_ (LXA_4_) and CMKLR1 (Chemokine-like receptor 1) for resolvin E1 (RvE1). LXA_4_ inhibits NK cell cytotoxicity and increases eosinophil-induced apoptosis by NK cells, and inhibits interleukin (IL)-13 release by ILC2s. RvE1 increases airway eosinophilia and antigen (Ag)-specific CD4^+^ T-cell clearance.

It is of interest that NK cells express pro-resolving receptors and that binding to pro-resolving mediators can modulate NK cell effector functions (fig. 2). NK cells were identified to express ALX/FPR2, a receptor for the pro-resolving mediator LXA_4_, and NK cells from subjects with severe asthma have increased ALX/FPR2 expression [31]. LXA_4_ can inhibit in a dose-dependent fashion the cytotoxic activity of human NK cells against K562 target cells assayed *in vitro* [106]. Moreover, when NK cells are exposed to LXA_4_, the cells display an increase in NK cell-mediated apoptosis of both eosinophils and neutrophils [31]. NK cells also express CMKLR1 (Chemokine-like receptor 1), also known as ChemR23 (Chemerin Receptor 23) [31, 107], a receptor for the pro-resolving mediator RvE1. RvE1 has been shown to be a potent pro-resolving mediator for allergic airway inflammation [60]. In a murine model of allergic lung inflammation, NK cell depletion markedly impaired RvE1’s protective actions [97]. Moreover, RvE1 regulates NK cell cytotoxicity *in vitro* [97].

### Type 2 ILCs

In addition to NK cells, the ILC family also includes type 2 ILCs (ILC2s). ILCs have similar morphologies to T-cells but do not express T- or B-cell antigen receptors or markers of other lineages. They are involved in responses to helminth infection and murine models of allergic lung inflammation, and can respond to the epithelial-derived cytokines IL-25 (also known as IL-17E), IL-33 and thymic stromal lymphopoietin. In an antigen-independent manner, ILC2s can generate the cytokines IL-5 and IL-13 that were previously linked to T-helper type 2 (Th2) lymphocytes. ILC2s were recently identified in humans as a population of

Lin^−^ cells expressing IL-7R, CRTH2 (a chemoattractant receptor for prostaglandin D_2_ that is also expressed on Th2 cells), and the common NK cell marker CD161 [108].

ILC2s are instrumental in several models of experimental asthma where they significantly contribute to the production of IL-5 and IL-13, and to AHR development (fig. 2) [109–114]. ILC2s are present in the blood of asthmatic patients [31]. Human ILC2s express the pro-resolving receptors ALX/FPR2, and LXA_4_ prevents prostaglandin D_2_-stimulated release of IL-13 from ILC2s.

In some cases, ILC2 can help restore lung-tissue homeostasis after influenza infection in a murine model. In a study by Monticelli *et al.* [115] following influenza virus infection, ILC2 depletion led to impaired lung function and tissue repair, along with increased permeability of the lung epithelial barrier. It was shown that ILC2s produce a factor from the epidermal growth factor family linked to tissue remodelling and repair in asthma, known as amphiregulin. Administration of amphiregulin restored epithelial cell integrity, airway remodelling, and lung function [115]. In line with these findings, the IL-33–ILC2–IL-13 axis has been reported to mediate tissue repair functions in a mouse model of biliary injury by promoting epithelial restoration [116].

Thus, ILC2 may both promote inflammatory lung disease and also restore airway epithelial cell integrity after injury. While these two functions of ILC2s may appear contradictory, the homeostatic *versus* the pathological role of ILCs may be similar to the contrasting roles of several other immune cell types. The context in which ILC2s function may determine whether the cells are beneficial (enhancing epithelial cell integrity) or detrimental (causing airway inflammation and airway hyperresponsiveness). ILCs may have evolved to respond rapidly during viral infections and when activated in the absence of appropriate regulation, ILC2s may cause disease, such as airway inflammation and AHR.

### Emerging roles of eosinophils and eosinophil-derived pro-resolving lipid mediators in asthma

**FIGURE 3:**
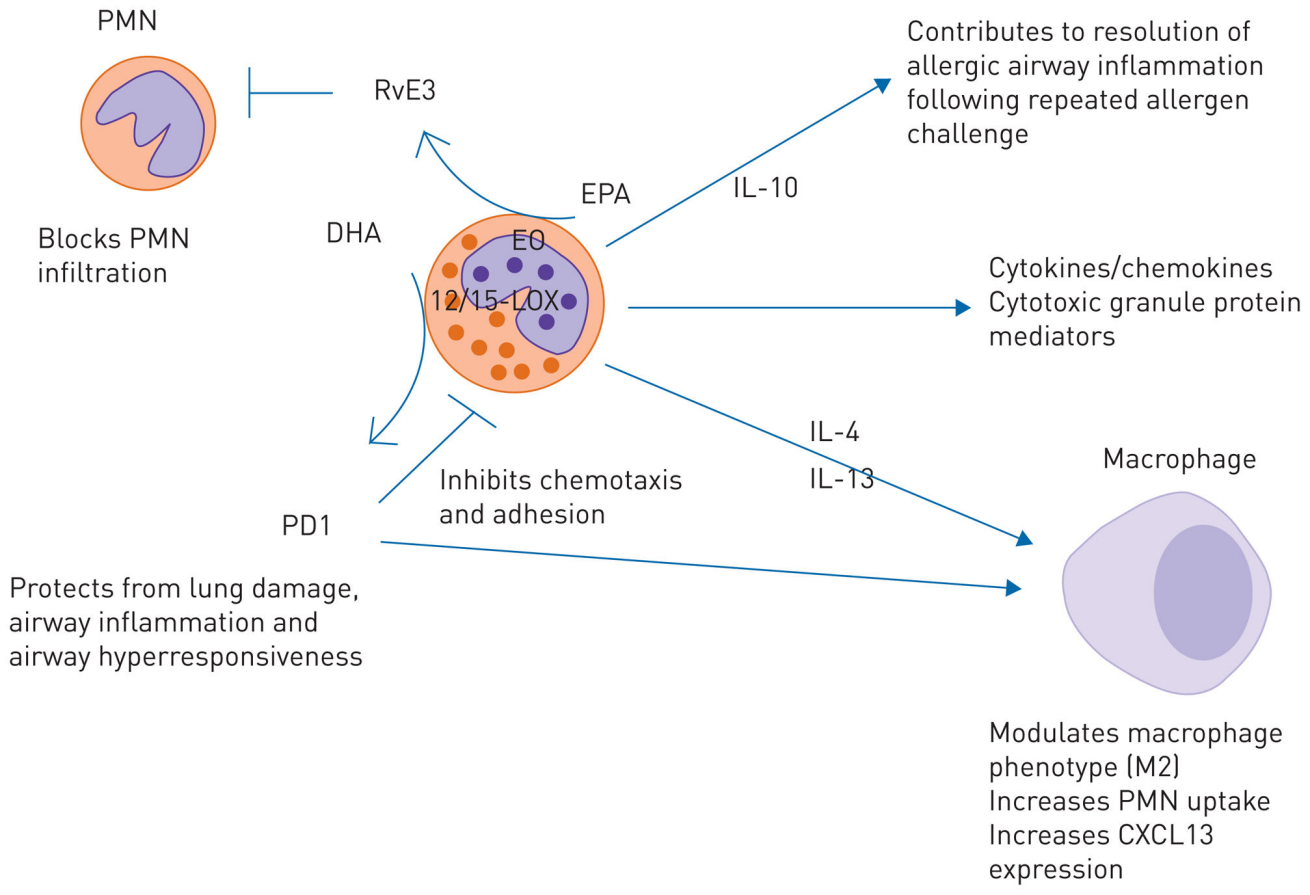
Key functions of eosinophils (Eo) in resolution of inflammation in asthma. Eosinophils secrete a wide array of cytotoxic and pro-inflammatory mediators. In addition, eosinophils may contribute to resolution of inflammation in asthma by producing pro-resolving lipid mediators (PD1 and RvE3), by secreting interleukin (IL)-10 and by promoting alternative macrophage activation in an IL-4 and IL-13 dependent manner. PMN: polymorphonuclear leukocyte; PD1: protectin D1; RvE3: resolvin E3; EPA: eicosapentaenoic acid; DHA: docosahexaenoic acid; LOX: lipoxygenase.

Eosinophils are associated with the pathogenesis of asthma, and their accumulation in the lungs is often regarded as a defining feature of allergic asthma in humans and in animal models [117]. It is assumed that eosinophils are recruited to the lungs by Th2 cells as end-stage effector cells, because of their ability to secrete a wide array of cytotoxic and pro-inflammatory mediators. For example, eosinophils can serve as major effector cells inducing tissue damage and dysfunction by releasing an array of cytotoxic granule cationic proteins including major basic protein, eosinophil cationic protein and eosinophil-derived neurotoxin (fig. 3) [118]. Nevertheless, the role of eosinophils in specific features of asthma has been controversial in several experimental and clinical studies [119–121]. Recent work showed that eosinophils are also able to contribute to the resolution of acute inflammation. In a murine model of self-limited zymosan-induced peritonitis, 12/15-LOX-expressing eosinophils were recruited to the inflamed loci during the resolution phase of the acute inflammatory response and were shown to generate pro-resolving lipid mediators, including PD1 [122]. In this nonallergen model, eosinophil-derived PD1 was shown to induce macrophage activity to clear apoptotic neutrophils from the site of inflammation. Eosinophils can also promote resolution in murine zymosan-induced inflammation by regulating the expression of macrophage CXCL13 through the control of the 12/15-LOX-derived mediator, LXA_4_ [123].

Interestingly, in a murine model of allergic lung inflammation, PD1, administered before aeroallergen challenge, reduced airway inflammation and dampened airway hyperresponsiveness [77]. In addition, in this study levels of PD1 were significantly lower in exhaled breath condensates from subjects with asthma exacerbations when compared with healthy subjects. PD1 has been confirmed as one of the main anti-inflammatory and pro-resolving molecules synthesised by human eosinophils [94]. PD1 is an autacoid regulator of eosinophils, and suppresses in nanomolar concentrations eosinophil chemotaxis induced by CCL11/eotaxin-1 or 5-oxo-eicosatetraenoic acid and modulates the expression of the adhesion molecules CD11b and L-selectin; although it has no significant effects on eosinophil degranulation, superoxide anion generation or survival (fig. 3). When compared with the cells harvested from healthy subjects, biosynthesis of PD1 is decreased in severe asthma [94].

Eosinophils are also able to convert 18-hydroperoxyeicosapentaenoic acid (HEPE) into 17,18-diHEPE, known as RvE3, *via* the 12/15-LOX pathway [52]. RvE3 displays potent anti-inflammatory activity by blocking PMN infiltration in acute peritonitis (fig. 3) [52].

The role of eosinophils in the resolution of inflammation probably extends beyond production of pro-resolving lipid mediators and includes pathways resulting from interactions with other pro-inflammatory and resident cells in the lung. Recent work showed that eosinophils are able to contribute to the resolution of lung-allergic responses following repeated allergen challenge in a murine model by producing IL-10, a potent anti-inflammatory cytokine [124]. *In vitro,* eosinophils have the potential to polarise macrophages through IL-4/13 release to an M2 phenotype, the precursor to resolution macrophages (*i.e.* increased phagocytic activity and production of resolving lipid mediators) [125, 126]. Eosinophils were also shown to promote alternatively activated macrophages in other disease models (fig. 3) [127, 128].

## Therapeutic implications

For asthma, currently available anti-inflammatory agents or therapies under development (*e.g.* glucocorticoids and the biological therapeutics anti-IgE, IL-5 or IL-13) target pro-inflammatory mediator pathways [129]. While this strategy has been beneficial in some clinical conditions, long-term use of corticosteroids can be associated with significant side-effects and there remain substantial unmet clinical needs [130].

Anti-inflammation and pro-resolution are not synonymous. The definitions of these terms have important differences. Although anti-leukocyte actions are commonly considered anti-inflammatory, it is important to view the role of each cell type in this dynamic process of catabasis. Inhibition of neutrophil transmigration and activation is anti-inflammatory but can lead to immunosuppression that increases the host’s susceptibility to infection. By contrast, restitution of barrier integrity (endothelial, epithelial, or both); recruitment of monocytoid cells; and promotion of macrophage clearance of apoptotic cells, microbes and tissue debris are all pro-resolving responses, increasing host defence [8, 22]. Therefore, the identification of such endogenous anti-inflammatory and/or pro-resolution mechanisms is of wide interest.

Rather than blocking early or select pro-inflammatory mediators, an alternative therapeutic strategy might emphasise mimetics of lipoxins, resolvins, protectins, maresins or other natural counter-regulatory molecules that accelerate resolution of inflammation. Metabolically stable analogues of some of these compounds have been developed and display potent *in vivo* protective actions in several asthma model systems (table 2). Moreover, more severe variants of asthma are associated with diminished lipoxin biosynthesis compared with milder asthma (table 3) [79, 82], suggesting that the chronic inflammatory response in asthma may be due, in part, to defective generation of pro-resolving mediators leading to inadequate counter-regulation. Exogenous administration of this mediator may benefit these patients by inhibiting and resolving inflammation.

In a recent clinical study, a LXA_4_-based compound was tested for the topical treatment of infantile eczema [131]. Albeit in a small number of patients, the drug reduced the severity of eczema to a similar extent as steroid therapy in a double-blind placebo-controlled setting. No studies, to date, have been published examining the therapeutic effect of LXA_4_ in asthma.

## Conclusion

The resolution of inflammation is integral to the physiological response to tissue injury, infection and allergens or other noxious stimuli. Resolution is an active process with highly regulated cellular and biochemical events that are engaged to restore tissue function in health. Recent discoveries have highlighted that innate inflammatory cells have bimodal effector functions during the inflammatory response, including active roles during the resolution process. Several mediators displaying potent pro-resolving actions have recently been uncovered. LXA_4_, the lead member of this new class of pro-resolving mediators, has anti-inflammatory actions on ILC2 and pro-resolving actions through NK cells in asthma immunobiology. Eosinophils are also able to control crucial aspects of resolution through the generation of pro-resolving mediators. Uncontrolled asthma has been linked to a defect in the generation of specialised pro-resolving mediators, including LXA_4_ and PD1. Thus, bioactive stable analogue mimetics of these mediators that can harness endogenous resolution mechanisms for inflammation may offer new therapeutic strategies for asthma and airway inflammation associated diseases.

## Figures and Tables

**TABLE 1:** Cellular actions of specialised pro-resolving lipid mediators in innate immunity relevant to asthma

Mediator	Target cell	Action(s)	References
**Lipoxin A_4_**	Eosinophil	Inhibits migration and chemotaxis	[24, 25]
		Inhibits generation of eotaxin and IL-5	
	Neutrophil	Inhibit chemotaxis	[26–30]
		Inhibit trans-endothelial and trans-epithelial migration	
		Inhibit neutrophil–epithelial cell interactions	
		Inhibit superoxide anion generation	
		Inhibit degranulation	
	NK cell	Inhibits NK cell cytotoxicity	[31, 32]
		Increases granulocyte induced apoptosis	
	ILC2	Inhibits IL-13 release	[31]
	Monocyte	Stimulates chemotaxis and adhesion	[33–35]
		Inhibits peroxynitrite generation	
		Reduces IL-8 release by cells from individuals with asthma	
	Macrophage	Increases engulfment of apoptotic neutrophils	[36, 37]
	Dendritic cell	Inhibits IL-12 production	[38]
	Epithelial cell	Increases proliferation after acid injury	[39, 40]
		Inhibits cytokine release	
		Increases intracellular Ca^2+^	
	Endothelial cell	Stimulates protein kinase-dependent prostacyclin formation	[41–43]
		Blocks the generation of reactive oxygen species	
		Inhibits VEGF-induced endothelial-cell migration	
	Fibroblast	Inhibits IL-1β-induced IL-6, IL-8 and MMP3 production	[44, 45]
		Inhibits CTGF-induced proliferation	
	Smooth muscle	Inhibits LTC4-initiated migration	[46]
	Bronchial epithelial cell	Stimulates basal cell proliferation after acid injury	[39]
		Blocks IL-6 and IL-8 release	
**Resolvin E1**	Neutrophil	Inhibits trans-epithelial and trans-endothelial migration	[47, 48]
		Inhibition of superoxide generation	
	Macrophage	Stimulates nonphlogistic phagocytosis of apoptotic neutrophils	[49]
	Dendritic cell	Inhibits IL-12 production	[50, 51]
		Inhibits migration	
**Resolvin E3**	Neutrophil	Inhibits infiltration	[52]
**Resolvin D1**	Neutrophil	Inhibits transmigration	[53]
	Macrophage	Inhibits LPS-induced TNF release	[54, 55]
		Promotes phagocytosis of antigen	
**Protectin D1**	Neutrophil	Inhibits TNF-α and IFN-γ release	[56–58]
		Inhibits PMN transmigration	
		Upregulates CCR5 expression	
	Macrophage	Stimulates nonphlogistic phagocytosis of apoptotic neutrophils	[49]

IL: interleukin; NK: natural killer; ILC2: type 2 innate lymphoid cell; VEGF: vascular endothelial growth factor; MMP3: matrix metalloproteinase 3; CTGF: connective tissue growth factor; LTC4: leukotriene C4; LPS: lipopolysaccharide; TNF: tumor necrosis factor; IFN-γ: interferon-γ; PMN: polymorphonuclear leukocyte; CCR5: CC-chemokine receptor 5.

**TABLE 2:** Biological activities of pro-resolving lipid mediators in asthma and allergic disease models

Mediator	Action	References
**Lipoxins**	Inhibits airway hyperresponsiveness and pulmonary inflammation	[68, 76]
**Resolvin E1**	Reduces airway inflammation; stimulates LXA_4_ production; and reduces SRS-A	[60]
**Resolvin D1**	Inhibits airway hyperresponsiveness and pulmonary inflammation	[54]
**Protectin D1**	Protects from lung damage, airway inflammation and airway hyperresponsiveness	[77]

LXA_4_: lipoxin A_4_; SRS-A: slow-reacting substance of anaphylaxis.

**TABLE 3:** Defects of pro-resolving mediators in asthma

Mediator		Population	References
**Lipoxin A4 (LXA_4_)**	Aspirin-tolerant asthmatics generate more lipoxins than aspirin-intolerant asthmatics	Adults	[83]
	Higher urinary aspirin-triggered lipoxin levels in aspirin-tolerant asthma than in aspirin-intolerant asthma	Adults	[84]
	Diminished lipoxin biosynthesis in severe asthma	Adults	[82]
	LXA_4_ defect in induced sputum in severe asthma	Adults	[33]
	Severe asthma is associated with a loss of LXA_4_ in induced sputum	Adults	[80]
	LXA_4_ generation is decreased in aspirin-sensitive patients in nasal lavage after aspirin nasal challenge	Adults	[85]
	LXA_4_ levels in asthma show a relationship with disease severity and aspirin sensitivity	Adults	[86]
	Airway LXA_4_ generation and LXA_4_ receptor expression are decreased in severe asthma	Adults	[79]
	The role of LXA_4_ in exercise-induced bronchoconstriction in asthma	Adults	[87]
	LXA_4_ biosynthesis is decreased in severe asthma alveolar macrophages	Adults	[88]
	Reversed changes of LXA_4_ and leukotrienes in children with asthma of different severity degree	Children	[89]
	LXA_4_ is decreased in the EBC of children recovering from status asthmaticus	Children	[90]
	LXA_4_ levels are lower in severe asthma and correlate negatively to lung function	Adults	[91]
	LXA_4_ levels in EBC are lower in moderate-to-severe asthma than in mild asthma	Adults	[92]
	Decreased levels of LXA_4_ in wheezy infants (blood)	Children	[93]
**Protectin D1 (PD1)**	PD1 is diminished in EBC from subjects with asthma exacerbation	Adults	[77]
	Impaired PD1 production in eosinophils from subjects with severe asthma	Adults	[94]

EBC: exhaled breath condensate.

## References

[1] Djukanović R, Roche WR, Wilson JW (1990). Mucosal inflammation in asthma. Am Rev Respir Dis.

[2] Lloyd CM, Hessel EM (2010). Functions of T cells in asthma: more than just T_H_2 cells. Nature Rev Immunol.

[3] Holgate ST (2012). Innate and adaptive immune responses in asthma. Nat Med.

[4] Deckers J, Branco Madeira F, Hammad H (2013). Innate immune cells in asthma. Trends Immunol.

[5] Busse WW, Lemanske RF (2001). Asthma. N Engl J Med.

[6] Gilroy DW, Lawrence T, Perretti M (2004). Inflammatory resolution: new opportunities for drug discovery. Nature Rev Drug Discov.

[7] Serhan CN, Chiang N, Van Dyke TE (2008). Resolving inflammation: dual anti-inflammatory and pro-resolution lipid mediators. Nature Rev Immunol.

[8] Levy BD, Serhan CN (2014). Resolution of acute inflammation in the lung. Annu Rev Physiol.

[9] Lawrence T, Willoughby DA, Gilroy DW (2002). Anti-inflammatory lipid mediators and insights into the resolution of inflammation. Nature Rev Immunol.

[10] Larsen GL, Henson PM (1983). Mediators of inflammation. Annu Rev Immunol.

[11] Nathan C, Ding A (2010). Nonresolving inflammation. Cell.

[12] Serhan CN, Savill J (2005). Resolution of inflammation: the beginning programs the end. Nat Immunol.

[13] Henson PM (2005). Dampening inflammation. Nat Immunol.

[14] Savill J (1998). Apoptosis. Phagocytic docking without shocking. Nature.

[15] Wynn TA, Chawla A, Pollard JW (2013). Macrophage biology in development, homeostasis and disease. Nature.

[16] Fadok VA, Bratton DL, Konowal A (1998). Macrophages that have ingested apoptotic cells *in vitro* inhibit proinflammatory cytokine production through autocrine/paracrine mechanisms involving TGF-β, PGE2, and PAF. J Clin Invest.

[17] Freire-de-Lima CG, Xiao YQ, Gardai SJ (2006). Apoptotic cells, through transforming growth factor-beta, coordinately induce anti-inflammatory and suppress pro-inflammatory eicosanoid and NO synthesis in murine macrophages. J Biol Chem.

[18] Serhan CN (2007). Resolution phase of inflammation: novel endogenous anti-inflammatory and proresolving lipid mediators and pathways. Annu Rev Immunol.

[19] Planaguma A, Levy BD (2008). Uncontrolled airway inflammation in lung disease represents a defect in counter-regulatory signaling. Future Lipidol.

[20] Serhan CN, Clish CB, Brannon J (2000). Novel functional sets of lipid-derived mediators with antiinflammatory actions generated from omega-3 fatty acids *via* cyclooxygenase 2-nonsteroidal antiinflammatory drugs and transcellular processing. J Exp Med.

[21] Serhan CN, Hong S, Gronert K (2002). Resolvins: a family of bioactive products of omega-3 fatty acid transformation circuits initiated by aspirin treatment that counter proinflammation signals. J Exp Med.

[22] Serhan CN, Brain SD, Buckley CD (2007). Resolution of inflammation: state of the art, definitions and terms. FASEB J.

[23] Levy BD, Clish CB, Schmidt B (2001). Lipid mediator class switching during acute inflammation: signals in resolution. Nat Immunol.

[24] Soyombo O, Spur BW, Lee TH (1994). Effects of lipoxin A4 on chemotaxis and degranulation of human eosinophils stimulated by platelet-activating factor and N-formyl-L-methionyl-L-leucyl-L-phenylalanine. Allergy.

[25] Bandeira-Melo C, Serra MF, Diaz BL (2000). Cyclooxygenase-2-derived prostaglandin E2 and lipoxin A4 accelerate resolution of allergic edema in Angiostrongylus costaricensis-infected rats: relationship with concurrent eosinophilia. J Immunol.

[26] Serhan CN, Maddox JF, Petasis NA (1995). Design of lipoxin A4 stable analogs that block transmigration and adhesion of human neutrophils. Biochemistry.

[27] Colgan SP, Serhan CN, Parkos CA (1993). Lipoxin A4 modulates transmigration of human neutrophils across intestinal epithelial monolayers. J Clin Invest.

[28] Papayianni A, Serhan CN, Brady HR (1996). Lipoxin A4 and B4 inhibit leukotriene-stimulated interactions of human neutrophils and endothelial cells. J Immunol.

[29] Levy BD, Fokin VV, Clark JM (1999). Polyisoprenyl phosphate (PIPP) signaling regulates phospholipase D activity: a ‘[stop’ signaling switch for aspirin-triggered lipoxin A4. FASEB J.

[30] Lee TH, Horton CE, Kyan-Aung U (1989). Lipoxin A4 and lipoxin B4 inhibit chemotactic responses of human neutrophils stimulated by leukotriene B4 and N-formyl-L-methionyl-L-leucyl-L-phenylalanine. Clin Sci.

[31] Barnig C, Cernadas M, Dutile S (2013). Lipoxin A4 regulates natural killer cell and type 2 innate lymphoid cell activation in asthma. Sci Transl Med.

[32] Ramstedt U, Serhan CN, Nicolaou KC (1987). Lipoxin A-induced inhibition of human natural killer cell cytotoxicity: studies on stereospecificity of inhibition and mode of action. J Immunol.

[33] Bonnans C, Vachier I, Chavis C (2002). Lipoxins are potential endogenous antiinflammatory mediators in asthma. Am J Respir Crit Care Med.

[34] Maddox JF, Serhan CN (1996). Lipoxin A4 and B4 are potent stimuli for human monocyte migration and adhesion: selective inactivation by dehydrogenation and reduction. J Exp Med.

[35] József L, Zouki C, Petasis NA (2002). Lipoxin A_4_ and aspirin-triggered 15-epi-lipoxin A_4_ inhibit peroxynitrite formation, NF-κB and AP-1 activation, and IL-8 gene expression in human leukocytes. Proc Natl Acad Sci USA.

[36] Godson C, Mitchell S, Harvey K (2000). Cutting edge: lipoxins rapidly stimulate nonphlogistic phagocytosis of apoptotic neutrophils by monocyte-derived macrophages. J Immunol.

[37] Mitchell S, Thomas G, Harvey K (2002). Lipoxins, aspirin-triggered epi-lipoxins, lipoxin stable analogues, and the resolution of inflammation: stimulation of macrophage phagocytosis of apoptotic neutrophils *in vivo*. J Am Soc Nephrol.

[38] Aliberti J, Hieny S, Reis e Sousa C (2002). Lipoxin-mediated inhibition of IL-12 production by DCs: a mechanism for regulation of microbial immunity. Nat Immunol.

[39] Bonnans C, Fukunaga K, Levy MA (2006). Lipoxin A_4_ regulates bronchial epithelial cell responses to acid injury. Am J Pathol.

[40] Bonnans C, Mainprice B, Chanez P (2003). Lipoxin A_4_ stimulates a cytosolic Ca^2+^ increase in human bronchial epithelium. J Biol Chem.

[41] Brezinski ME, Gimbrone MA, Nicolaou KC (1989). Lipoxins stimulate prostacyclin generation by human endothelial cells. FEBS Lett.

[42] Nascimento-Silva V, Arruda MA, Barja-Fidalgo C (2007). Aspirin-triggered lipoxin A4 blocks reactive oxygen species generation in endothelial cells: a novel antioxidative mechanism. Thromb Haemost.

[43] Cezar-de-Mello PF, Nascimento-Silva V, Villela CG (2006). Aspirin-triggered lipoxin A4 inhibition of VEGF-induced endothelial cell migration involves actin polymerization and focal adhesion assembly. Oncogene.

[44] Sodin-Semrl S, Taddeo B, Tseng D (2000). Lipoxin A_4_ inhibits IL-1 β-induced IL-6, IL-8, and matrix metalloproteinase-3 production in human synovial fibroblasts and enhances synthesis of tissue inhibitors of metalloproteinases. J Immunol.

[45] Wu SH, Wu XH, Lu C (2006). Lipoxin A4 inhibits proliferation of human lung fibroblasts induced by connective tissue growth factor. Am J Respir Cell Mol Biol.

[46] Parameswaran K, Radford K, Fanat A (2007). Modulation of human airway smooth muscle migration by lipid mediators and Th-2 cytokines. Am J Respir Cell Mol Biol.

[47] Campbell EL, Louis NA, Tomassetti SE (2007). Resolvin E1 promotes mucosal surface clearance of neutrophils: a new paradigm for inflammatory resolution. FASEB J.

[48] Hasturk H, Kantarci A, Ohira T (2006). RvE1 protects from local inflammation and osteoclast-mediated bone destruction in periodontitis. FASEB J.

[49] Schwab JM, Chiang N, Arita M (2007). Resolvin E1 and protectin D1 activate inflammation-resolution programmes. Nature.

[50] Arita M, Bianchini F, Aliberti J (2005). Stereochemical assignment, antiinflammatory properties, and receptor for the omega-3 lipid mediator resolvin E1. J Exp Med.

[51] Arita M, Yoshida M, Hong S (2005). Resolvin E1, an endogenous lipid mediator derived from omega-3 eicosapentaenoic acid, protects against 2,4,6-trinitrobenzene sulfonic acid-induced colitis. Proc Natl Acad Sci USA.

[52] Isobe Y, Arita M, Matsueda S (2012). Identification and structure determination of novel anti-inflammatory mediator resolvin E3, 17,18-dihydroxyeicosapentaenoic acid. J Biol Chem.

[53] Sun YP, Oh SF, Uddin J (2007). Resolvin D1 and its aspirin-triggered 17R epimer. Stereochemical assignments, anti-inflammatory properties, and enzymatic inactivation. J Biol Chem.

[54] Rogerio AP, Haworth O, Croze R (2012). Resolvin D1 and aspirin-triggered resolvin D1 promote resolution of allergic airways responses. J Immunol.

[55] Duffield JS, Hong S, Vaidya VS (2006). Resolvin D series and protectin D1 mitigate acute kidney injury. J Immunol.

[56] Ariel A, Li PL, Wang W (2005). The docosatriene protectin D1 is produced by TH2 skewing and promotes human T cell apoptosis *via* lipid raft clustering. J Biol Chem.

[57] Bannenberg GL, Chiang N, Ariel A (2005). Molecular circuits of resolution: formation and actions of resolvins and protectins. J Immunol.

[58] Ariel A, Fredman G, Sun YP (2006). Apoptotic neutrophils and T cells sequester chemokines during immune response resolution through modulation of CCR5 expression. Nat Immunol.

[59] Serhan CN, Hamberg M, Samuelsson B (1984). Lipoxins: novel series of biologically active compounds formed from arachidonic acid in human leukocytes. Proc Natl Acad Sci USA.

[60] Haworth O, Cernadas M, Yang R (2008). Resolvin E1 regulates interleukin 23, interferon-γ and lipoxin A4 to promote the resolution of allergic airway inflammation. Nat Immunol.

[61] Hunter JA, Finkbeiner WE, Nadel JA (1985). Predominant generation of 15-lipoxygenase metabolites of arachidonic acid by epithelial cells from human trachea. Proc Natl Acad Sci USA.

[62] Levy BD, Romano M, Chapman HA (1993). Human alveolar macrophages have 15-lipoxygenase and generate 15 (S)-hydroxy-5,8,11-cis-13-trans-eicosatetraenoic acid and lipoxins. J Clin Invest.

[63] Serhan CN, Hirsch U, Palmblad J (1987). Formation of lipoxin A by granulocytes from eosinophilic donors. FEBS Lett.

[64] Shannon VR, Chanez P, Bousquet J (1993). Histochemical evidence for induction of arachidonate 15-lipoxygenase in airway disease. Am Rev Respir Dis.

[65] Serhan CN (2005). Lipoxins and aspirin-triggered 15-epi-lipoxins are the first lipid mediators of endogenous anti-inflammation and resolution. Prostaglandins Leukot Essent Fatty Acids.

[66] Clària J, Serhan CN (1995). Aspirin triggers previously undescribed bioactive eicosanoids by human endothelial cell-leukocyte interactions. Proc Natl Acad Sci USA.

[67] Chiang N, Serhan CN, Dahlen SE (2006). The lipoxin receptor ALX: potent ligand-specific and stereoselective actions *in vivo*. Pharmacol Rev.

[68] Levy BD, De Sanctis GT, Devchand PR (2002). Multi-pronged inhibition of airway hyper-responsiveness and inflammation by lipoxin A_4_. Nat Med.

[69] Schaldach CM, Riby J, Bjeldanes LF (1999). Lipoxin A4: a new class of ligand for the Ah receptor. Biochemistry.

[70] Schwartz J, Weiss ST (1994). The relationship of dietary fish intake to level of pulmonary function in the first National Health and Nutrition Survey (NHANES I). Eur Respir J.

[71] Bannenberg G, Serhan CN (2010). Specialized pro-resolving lipid mediators in the inflammatory response: an update. Biochim Biophys Acta.

[72] Serhan CN (2014). Pro-resolving lipid mediators are leads for resolution physiology. Nature.

[73] Cash JL, Hart R, Russ A (2008). Synthetic chemerin-derived peptides suppress inflammation through ChemR23. J Exp Med.

[74] Samson M, Edinger AL, Stordeur P (1998). ChemR23, a putative chemoattractant receptor, is expressed in monocyte-derived dendritic cells and macrophages and is a coreceptor for SIV and some primary HIV-1 strains. Eur J Immunol.

[75] Wittamer V, Franssen JD, Vulcano M (2003). Specific recruitment of antigen-presenting cells by chemerin, a novel processed ligand from human inflammatory fluids. J Exp Med.

[76] Levy BD, Lukacs NW, Berlin AA (2007). Lipoxin A4 stable analogs reduce allergic airway responses *via* mechanisms distinct from CysLT1 receptor antagonism. FASEB J.

[77] Levy BD, Kohli P, Gotlinger K (2007). Protectin D1 is generated in asthma and dampens airway inflammation and hyperresponsiveness. J Immunol.

[78] Lee TH, Crea AE, Gant V (1990). Identification of lipoxin A4 and its relationship to the sulfidopeptide leukotrienes C4, D4, and E4 in the bronchoalveolar lavage fluids obtained from patients with selected pulmonary diseases. Am Rev Respir Dis.

[79] Planagumà A, Kazani S, Marigowda G (2008). Airway lipoxin A4 generation and lipoxin A4 receptor expression are decreased in severe asthma. Am J Respir Crit Care Med.

[80] Vachier I, Bonnans C, Chavis C (2005). Severe asthma is associated with a loss of LX4, an endogenous anti-inflammatory compound. J Allergy Clin Immunol.

[81] Christie PE, Spur BW, Lee TH (1992). The effects of lipoxin A4 on airway responses in asthmatic subjects. Am Rev Respir Dis.

[82] Levy BD, Bonnans C, Silverman ES (2005). Diminished lipoxin biosynthesis in severe asthma. Am J Respir Crit Care Med.

[83] Sanak M, Levy BD, Clish CB (2000). Aspirin-tolerant asthmatics generate more lipoxins than aspirin-intolerant asthmatics. Eur Respir J.

[84] Yamaguchi H, Higashi N, Mita H (2011). Urinary concentrations of 15-epimer of lipoxin A_4_ are lower in patients with aspirin-intolerant compared with aspirin-tolerant asthma. Clin Exp Allergy.

[85] Kupczyk M, Antczak A, Kuprys-Lipinska I (2009). Lipoxin A4 generation is decreased in aspirin-sensitive patients in lysine-aspirin nasal challenge *in vivo* model. Allergy.

[86] Celik GE, Erkekol FO, Misirligil Z (2007). Lipoxin A4 levels in asthma: relation with disease severity and aspirin sensitivity. Clin Exp Allergy.

[87] Tahan F, Saraymen R, Gumus H (2008). The role of lipoxin A4 in exercise-induced bronchoconstriction in asthma. J Asthma.

[88] Bhavsar PK, Levy BD, Hew MJ (2010). Corticosteroid suppression of lipoxin A4 and leukotriene B4 from alveolar macrophages in severe asthma. Respir Res.

[89] Wu SH, Yin PL, Zhang YM (2010). Reversed changes of lipoxin A4 and leukotrienes in children with asthma in different severity degree. Pediatr Pulmonol.

[90] Hasan RA, O’Brien E, Mancuso P (2012). Lipoxin A_4_ and 8-isoprostane in the exhaled breath condensate of children hospitalized for status asthmaticus. Pediatr Crit Care Med.

[91] Kazani S, Planaguma A, Ono E (2013). Exhaled breath condensate eicosanoid levels associate with asthma and its severity. J Allergy Clin Immunol.

[92] Fritscher LG, Post M, Rodrigues MT (2012). Profile of eicosanoids in breath condensate in asthma and COPD. J Breath Res.

[93] Eke Gungor H, Tahan F, Gokahmetoglu S (2014). Decreased levels of lipoxin A4 and annexin A1 in wheezy infants. Int Arch Allergy Immunol.

[94] Miyata J, Fukunaga K, Iwamoto R (2013). Dysregulated synthesis of protectin D1 in eosinophils from patients with severe asthma. J Allergy Clin Immunol.

[95] Bilal S, Haworth O, Wu L (2011). Fat-1 transgenic mice with elevated omega-3 fatty acids are protected from allergic airway responses. Biochim Biophys Acta.

[96] Aoki H, Hisada T, Ishizuka T (2008). Resolvin E1 dampens airway inflammation and hyperresponsiveness in a murine model of asthma. Biochem Biophys Res Commun.

[97] Haworth O, Cernadas M, Levy BD (2011). NK cells are effectors for resolvin E1 in the timely resolution of allergic airway inflammation. J Immunol.

[98] Diefenbach A, Colonna M, Koyasu S (2014). Development, differentiation, and diversity of innate lymphoid cells. Immunity.

[99] Vivier E, Raulet DH, Moretta A (2011). Innate or adaptive immunity? The example of natural killer cells. Science.

[100] Karimi K, Forsythe P (2013). Natural killer cells in asthma. Front Immunol.

[101] Korsgren M, Persson CG, Sundler F (1999). Natural killer cells determine development of allergen-induced eosinophilic airway inflammation in mice. J Exp Med.

[102] Ple C, Barrier M, Amniai L (2010). Natural killer cells accumulate in lung-draining lymph nodes and regulate airway eosinophilia in a murine model of asthma. Scand J immunol.

[103] Thorén FB, Riise RE, Ousbäck J (2012). Human NK cells induce neutrophil apoptosis *via* an NKp46- and Fas-dependent mechanism. J Immunol.

[104] Awad A, Yassine H, Barrier M (2014). Natural killer cells induce eosinophil activation and apoptosis. PLoS One.

[105] Woolley KL, Gibson PG, Carty K (1996). Eosinophil apoptosis and the resolution of airway inflammation in asthma. Am J Respir Crit Care Med.

[106] Ramstedt U, Ng J, Wigzell H (1985). Action of novel eicosanoids lipoxin A and B on human natural killer cell cytotoxicity: effects on intracellular cAMP and target cell binding. J Immunol.

[107] Parolini S, Santoro A, Marcenaro E (2007). The role of chemerin in the colocalization of NK and dendritic cell subsets into inflamed tissues. Blood.

[108] Mjösberg JM, Trifari S, Crellin NK (2011). Human IL-25- and IL-33-responsive type 2 innate lymphoid cells are defined by expression of CRTH2 and CD161. Nat Immunol.

[109] Barlow JL, Bellosi A, Hardman CS (2012). Innate IL-13-producing nuocytes arise during allergic lung inflammation and contribute to airways hyperreactivity. J Allergy Clin Immunol.

[110] Halim TY, Krauss RH, Sun AC (2012). Lung natural helper cells are a critical source of Th2 cell-type cytokines in protease allergen-induced airway inflammation. Immunity.

[111] Liang HE, Reinhardt RL, Bando JK (2012). Divergent expression patterns of IL-4 and IL-13 define unique functions in allergic immunity. Nat Immunol.

[112] Chang YJ, Kim HY, Albacker LA (2011). Innate lymphoid cells mediate influenza-induced airway hyper-reactivity independently of adaptive immunity. Nat Immunol.

[113] Klein Wolterink RG, Kleinjan A, van Nimwegen M (2012). Pulmonary innate lymphoid cells are major producers of IL-5 and IL-13 in murine models of allergic asthma. Eur J Immunol.

[114] Kim HY, Chang YJ, Subramanian S (2012). Innate lymphoid cells responding to IL-33 mediate airway hyperreactivity independently of adaptive immunity. J Allergy Clin Immunol.

[115] Monticelli LA, Sonnenberg GF, Abt MC (2011). Innate lymphoid cells promote lung-tissue homeostasis after infection with influenza virus. Nat Immunol.

[116] Li J, Razumilava N, Gores GJ (2014). Biliary repair and carcinogenesis are mediated by IL-33-dependent cholangiocyte proliferation. J Clin Invest.

[117] Fulkerson PC, Rothenberg ME (2013). Targeting eosinophils in allergy, inflammation and beyond. Nature Rev Drug Discov.

[118] Rothenberg ME, Hogan SP (2006). The eosinophil. Annu Rev Immunol.

[119] Fattouh R, Al-Garawi A, Fattouh M (2011). Eosinophils are dispensable for allergic remodeling and immunity in a model of house dust mite-induced airway disease. Am J Respir Crit Care Med.

[120] Leckie MJ (2003). Anti-interleukin-5 monoclonal antibodies: preclinical and clinical evidence in asthma models. Am J Respir Med.

[121] Flood-Page P, Swenson C, Faiferman I (2007). A study to evaluate safety and efficacy of mepolizumab in patients with moderate persistent asthma. Am J Respir Crit Care Med.

[122] Yamada T, Tani Y, Nakanishi H (2011). Eosinophils promote resolution of acute peritonitis by producing proresolving mediators in mice. FASEB J.

[123] Tani Y, Isobe Y, Imoto Y (2014). Eosinophils control the resolution of inflammation and draining lymph node hypertrophy through the proresolving mediators and CXCL13 pathway in mice. FASEB J.

[124] Takeda K, Shiraishi Y, Ashino S (2014). Eosinophils contribute to the resolution of lung-allergic responses following repeated allergen challenge. J Allergy Clin Immunol.

[125] Stolarski B, Kurowska-Stolarska M, Kewin P (2010). IL-33 exacerbates eosinophil-mediated airway inflammation. J Immunol.

[126] Kurowska-Stolarska M, Stolarski B, Kewin P (2009). IL-33 amplifies the polarization of alternatively activated macrophages that contribute to airway inflammation. J Immunol.

[127] Wu D, Molofsky AB, Liang HE (2011). Eosinophils sustain adipose alternatively activated macrophages associated with glucose homeostasis. Science.

[128] Qiu Y, Nguyen KD, Odegaard JI (2014). Eosinophils and type 2 cytokine signaling in macrophages orchestrate development of functional beige fat. Cell.

[129] Pelaia G, Vatrella A, Maselli R (2012). The potential of biologics for the treatment of asthma. Nature Rev Drug Discov.

[130] Chung KF, Wenzel SE, Brozek JL (2014). International ERS/ATS guidelines on definition, evaluation and treatment of severe asthma. Eur Respir J.

[131] Wu SH, Chen XQ, Liu B (2013). Efficacy and safety of 15(R/S)-methyl-lipoxin A_4_ in topical treatment of infantile eczema. Br J Dermatol.

